# Key Challenges and Barriers to Digital Literacy for Older Adults: Scoping Review

**DOI:** 10.2196/80647

**Published:** 2026-03-16

**Authors:** Salman Khan, Sarah Webster, John Puxty, Madison Robertson

**Affiliations:** 1 Public Health Sciences, Queen's University, Kingston, ON, Canada; 2 Centre for Studies in Aging and Health, Department of Medicine, Queen's University, Etherington Hall, Room 3046, 94 Stuart Street, Kingston, ON, K7L 3N6, Canada, 1-343-364-0786

**Keywords:** digital literacy, older adults, digital divide, eHealth, internet, technology, digital inclusion

## Abstract

**Background:**

Despite rising internet use in Canada, older adults continue to face significant barriers in adopting and using digital technologies. Digital literacy among older adults extends beyond technical proficiency, encompassing adaptation to new technologies, overcoming age-related limitations, and addressing socioeconomic disparities. Limited digital skills hinder social participation, access to essential services, and engagement with eHealth technologies, exacerbating disparities in health outcomes.

**Objective:**

This scoping review aimed to identify key barriers to digital literacy among older adults, synthesizing evidence from existing literature to provide a comprehensive understanding of the challenges older adults face in adopting and utilizing digital technologies.

**Methods:**

A systematic search was conducted across the Ovid MEDLINE, PubMed, Web of Science, and EBSCOhost databases to identify studies published in the past 10 years that examined digital literacy barriers among older adults. Data extraction captured population characteristics, study context, research methods, and outcomes, which were thematically analyzed to identify overarching categories and themes.

**Results:**

A total of 22 studies met the inclusion criteria. Most studies used a qualitative design (n=16, 72.72%), with the remaining studies comprising 3 quantitative studies (13.63%) and 3 reviews (13.63%). Seven primary barriers to digital literacy among older adults were identified: (1) health barriers, (2) support networks, (3) convenience and ease of use, (4) knowledge and information, (5) perception barriers, (6) resource barriers, and (7) barriers for special populations. Within these themes, 18 associated categories provided a more detailed understanding of the challenges older adults face in adopting and using digital technologies.

**Conclusions:**

These findings demonstrate that digital literacy challenges among older adults are shaped by interacting individual, social, and contextual factors rather than isolated skill deficits. Barriers often co-occur, with health-related limitations, access to support, technology design, and resource constraints jointly influencing digital engagement. This review highlights the need for integrated and inclusive digital literacy strategies that address compounded barriers and reflect the diverse circumstances of older adults. Future research should focus on establishing consistent definitions of digital literacy, evaluating the long-term impact of digital inclusion initiatives, and addressing the needs of subpopulations experiencing heightened or intersecting barriers.

## Introduction

### Background

Canada, with its diverse and aging population, faces unique challenges and opportunities in promoting digital inclusion for older adults. The older adult population in Canada is expected to grow by 68% between 2017 and 2037; the number of individuals aged 75 and older will double by 2037 [1]. These numbers highlight the urgency of addressing the unique needs and challenges of this population. Despite the high overall internet penetration in the country, a significant digital divide persists among older adults. The term “digital divide” refers to the various social disparities in access to and use of digital tools and services, such as personal computers and smartphones [2].

While digital literacy is essential for everyone in today’s technology-driven world, it is particularly crucial for older adults. The term “digital literacy” encompasses the skills required to use the internet and information and communication technologies (ICT) effectively, such as computers, smartphones, and social media [2]. For older adults, digital literacy often extends beyond mere technical proficiency. This includes understanding and adapting to new technologies, as well as overcoming barriers related to age-specific physical and cognitive challenges and socioeconomic factors that can limit access to digital tools. Research findings have consistently identified that key barriers to internet use among older adults include age, education, income, and computer-related factors [2,3]. As the digital landscape continues to evolve—marked by the widespread adoption of smartphones, social media, and online services—understanding how digital literacy for older adults is defined and applied across various contexts has become increasingly critical.

In Canada, while internet use among older adults has increased from 32.2% in 2007 to 68.2% in 2016, substantial socioeconomic and access-related disparities persist [4]. Factors, such as preretirement exposure to technology, health status, education, living arrangements, and household income, significantly influence internet use among this demographic. Certain vulnerable populations are disproportionately affected by this digital divide. For example, older adults with higher education levels, better health, and those living with others are more likely to be digitally active, while those with less education, poorer health, and living alone are less likely to use the internet [4]. These disparities highlight the need for targeted digital literacy initiatives tailored to the needs of older adults. Addressing these unique barriers can enhance digital engagement, improve older adults’ overall well-being, and enable greater participation in our increasingly digital world.

### Benefits and Barriers to Digital Literacy

Achieving digital literacy is vital for social inclusion, accessing information, and managing health care through eHealth technologies [3]. However, older adults often face significant barriers to accessing and using digital technologies. Older adults represent a unique demographic in this regard due to factors such as age-related declines in physical and cognitive abilities, limited prior exposure to technology, and varying levels of education and income [2]. The lack of motivation and the belief that digital technologies are not relevant to their needs can further hinder older adults’ engagement with these technologies, particularly in the context of digital literacy acquisition [4].

eHealth, which refers to health-related electronic technologies, particularly the internet, is one area where digital literacy is crucial for older adults [4]. eHealth literacy involves the ability to seek, understand, and apply health information from electronic sources. As more health care services move online, including booking and attending virtual appointments, accessing test results, and managing prescriptions, eHealth is increasingly essential for older adults to maintain autonomy and access necessary care [2]. However, the adoption of eHealth technologies remains low among older adults due to barriers such as design issues, cost, and lack of awareness [5]. A systematic review conducted identified a gap in the literature on eHealth literacy among underserved populations, including older adults [6]. While specific to the United States, this study emphasized the need for targeted research and interventions to improve digital health literacy in these groups.

The advantages of digital literacy for older adults are numerous. With improved digital skills, older adults gain better access to essential information, including health care resources, and can strengthen their social ties, positively influencing their mental and emotional well-being [3]. Additionally, engaging with ICT can stimulate cognition by offering access to games, news, and educational opportunities, which may help delay cognitive decline [3]. By improving digital literacy, age-related disparities can be reduced, fostering connections across diverse groups and promoting knowledge exchange [3]. However, despite these significant benefits, the substantial disparities in digital literacy among older adults in Canada, exacerbated by various demographic factors, highlight an urgent need to systematically explore and identify the most effective strategies and best practices for mitigating these barriers. By doing so, we can ensure that older adults are better equipped to obtain these critical benefits.

Although several reviews have explored older adults’ engagement with digital technologies, most have focused on narrow subpopulations or specific digital domains. Prior syntheses have commonly examined digital health adoption, targeted specific older adult groups (eg, those with chronic conditions), or evaluated the impacts and barriers of particular digital literacy interventions, rather than conceptualizing digital literacy as a broader construct [7-10]. For example, Hepburn et al [11] reviewed barriers to digital health technology adoption among older adults with chronic diseases, emphasizing health-related contexts rather than general digital skill acquisition. Similarly, another review focused specifically on older adults’ use of eHealth technologies, without addressing the broader range of skills, contexts, and supports that constitute digital literacy more generally [12]. Further research is needed to examine digital literacy as a multidimensional construct. To date, no review has comprehensively synthesized barriers and facilitators to digital literacy among the general older adult population across contexts, underscoring the need for an updated and inclusive scoping review.

### Purpose

This scoping review synthesizes existing literature on barriers to digital literacy among older adults and addresses the question: *What are the key challenges and barriers to digital literacy among older adults across different settings?* Framed using the population, concept, and context framework, the population of interest is older adults, the concept is challenges and barriers to digital literacy, and the context includes any relevant setting (eg, community, health care, home). Challenges and barriers were conceptualized broadly to include individual, social, technological, and contextual factors, including health-related considerations where relevant. A scoping review was chosen to map the breadth of existing evidence, identify gaps, and include diverse study designs, which would not be feasible with a systematic or narrative review.

## Methods

### Overview

This scoping review was conducted and reported in accordance with the PRISMA-ScR (Preferred Reporting Items for Systematic Reviews and Meta-Analyses extension for Scoping Reviews) guidelines [13]. The PRISMA-ScR guided the review process, including the development of the search strategy, study selection, data charting, and synthesis of results (Checklist 1).

### Search Strategy

The search strategy for this scoping review involved a comprehensive and systematic approach to identify relevant studies using multiple databases. A librarian from Queen’s University was consulted for this review. The databases used for this review included Ovid MEDLINE, PubMed, Web of Science, and EBSCOhost. The initial search was conducted using Ovid MEDLINE and PubMed databases in June 2024, with an updated search completed in December 2025 of all 4 databases. Search strategies combined controlled vocabulary terms (eg, Medical Subject Headings [MeSH]) and keyword searching, using Boolean operators (AND/OR) to capture concepts related to digital literacy (eg, “digital literacy,” “digital divide,” “digital inclusion,” “computer literacy”), barriers and challenges (eg, “barriers,” “challenges”), and the population of interest (eg, “older adults,” “seniors”). Search terms were adapted for each database to align with database-specific indexing and syntax (Multimedia Appendix 1). The reference lists of all included studies were also hand-searched to identify additional relevant literature. The review was limited to studies published within the past 10 years (2014 to the date of the search) to ensure relevance to contemporary digital technologies and evolving conceptualizations of digital literacy.

### Eligibility Criteria

This review included studies that (1) were conducted in any region, (2) included participants aged 50 years and older, (3) directly or indirectly examined digital literacy (including eHealth digital literacy), explicitly using this term or closely related synonyms, and (4) reported barriers to digital literacy. The age threshold was chosen to ensure that no relevant studies were missed and to align with previous studies that have similarly defined older adults with this age range to capture a broader range of experiences and challenges associated with aging and technology use [14,15].

This scoping review included peer-reviewed studies encompassing qualitative, quantitative, and mixed methods research studies, including systematic reviews, meta-analyses, and scoping reviews. Non–peer-reviewed studies, opinion pieces, and noncredible sources were not considered. Additionally, only studies and documents available in English were included to facilitate accessibility and comprehension.

### Study or Source of Evidence Selection

The study selection process used a structured, multistep approach to ensure the inclusion of the most relevant and high-quality studies. All identified documents were imported into Covidence [16]. Duplicates were removed, and titles and abstracts were initially screened by the primary investigator against the predefined inclusion and exclusion criteria. To minimize selection bias, a secondary investigator independently reviewed all titles and abstracts, as well as the full texts of the included and excluded studies, to confirm consistency with the inclusion and exclusion criteria. Discrepancies were resolved through discussion or, if needed, consultation with a third independent investigator. Although formal interrater reliability statistics (eg, Cohen kappa) were not calculated, this double-review and resolution process provided a high degree of interrater reliability and ensured the accuracy and consistency of study selection. The results of this search were recorded using the PRISMA (Preferred Reporting Items for Systematic Reviews and Meta-Analyses) flow diagram [17].

### Data Extraction

Data extraction was conducted to capture all relevant information from the selected studies. Key details, such as population characteristics (eg, age, gender, socioeconomic status), study context (eg, geographic location, setting), research methods, and outcomes (eg, levels of digital literacy, barriers identified, effectiveness of interventions), were systematically extracted by the primary investigator. A secondary investigator reviewed all extracted information for accuracy. The extracted information was organized into an Excel sheet (2019, version 2407) to facilitate easy comparison and analysis.

### Data Analysis and Presentation

The synthesis of the extracted data followed a systematic, iterative process informed by thematic analysis as outlined by Braun and Clarke [18]. Initially, the findings were grouped into categories of statements that reflected similar meanings across studies. These categories were then further analyzed and synthesized into overarching themes representing different types of barriers to digital literacy among older adults. Throughout this process, all decisions regarding category formation, theme development, and coding were documented to maintain an audit trail, ensuring transparency and reproducibility. This approach allowed us to identify 7 key barriers and associated contextual factors, which are presented in a coherent narrative format highlighting the main findings and insights from the review. The methodological quality of the included studies was appraised using the Joanna Briggs Institute (JBI) critical appraisal tools, with the specific checklist applied according to each study’s design (eg, qualitative, quantitative, or review). Quality appraisal was conducted to support the interpretation of the findings rather than to exclude studies.

### Ethical Considerations

As this study is a scoping review of publicly available literature and does not involve human participants or identifiable data, ethics approval was not required in accordance with the Queen’s University Research Ethics Board. An a priori internal protocol was established and used to guide the conduct of this scoping review. In accordance with current methodological guidance (eg, PRISMA-ScR, JBI Manual), prospective registration is not required for scoping reviews. The review was conducted as planned, and no substantive deviations from the internal protocol were made.

## Results

### Overview

**Figure 1.**
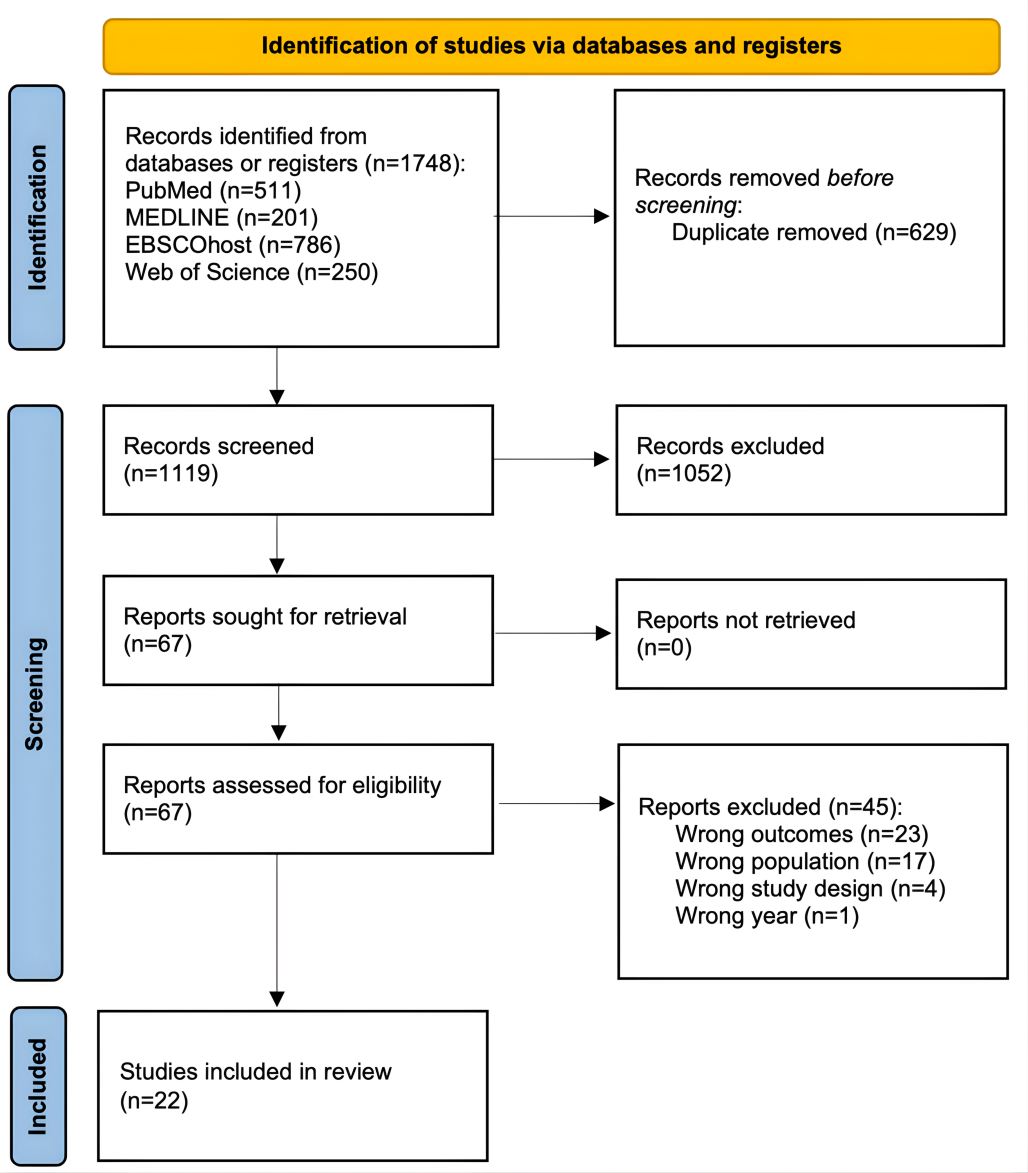
The PRISMA (Preferred Reporting Items for Systematic Reviews and Meta-Analyses) flow diagram [17].

This scoping review resulted in the identification of 1748 studies across 4 primary databases: PubMed (n=511), Ovid MEDLINE (n=201), Web of Science (n=786), and EBSCOhost (n=250). After electronically removing 629 duplicates, 1119 studies were screened against the inclusion and exclusion criteria at the title and abstract level. At this initial screening stage, 1052 studies were excluded. A total of 67 studies were assessed for eligibility through full-text review, leading to the exclusion of 45 studies for the following reasons: wrong outcomes (n=23), wrong population (n=17), wrong study design (n=4), and publication older than 10 years (n=1). Ultimately, 22 studies met the inclusion criteria and were included in the final review. A full outline of the search and selection process is provided in Figure 1.

### Study Demographics

Table 1 presents an overview of the characteristics of the studies included in this scoping review, highlighting the year of study publication, the region where the study was conducted, the study design, and the population age characteristics. Half (n=11, 50%) of the studies were recently published between 2022 and 2025. Geographically, the included studies were conducted across diverse global contexts. Four (18.18%) studies were conducted across multiple regions, while 7 (31.81%) studies were classified in the “Other” category, encompassing research conducted in South Korea, Malaysia, Norway, Turkey, Costa Rica, Spain, and China. A majority (n=16, 72.72%) of the studies used a qualitative study design, with 3 quantitative studies (13.63%) and 3 reviews (13.63%). A summary of each study’s characteristics is provided in Multimedia Appendix 2 [19-40].

**Table 1.** Study demographic information of the included studies (n=22).

Category	Count, n (%)
Year of study publication	
2022‐2025	11 (50)
2018‐2021	6 (27.27)
2014‐2017	5 (22.72)
Region where study was conducted	
Other (ie, South Korea, Malaysia, Norway, Turkey, Costa Rica, Spain, China)	7 (31.81)
Multiple regions or international	4 (18.18)
United States	3 (13.64)
United Kingdom	2 (9.09)
Canada	2 (9.09)
Singapore	2 (9.09)
Australia	2 (9.09)
Study design	
Qualitative	16 (72.72)
Review	3 (13.63)
Quantitative	3 (13.63)

### Quality Appraisal

Among the 22 studies included in this review, the overall methodological quality ranged from high to moderate quality, with no studies being excluded on the basis of their quality. Using the appropriate JBI critical appraisal tools [41], 6 studies were appraised as high quality, 12 as moderate quality, and 3 were moderate-unclear quality due to insufficient methodological reporting. The high-quality studies (n=6) were primarily qualitative interview or focus group designs that demonstrated strong alignment between research questions, methodology, and data analysis and reported strategies to enhance rigor, such as multiple coders, audit trails, and triangulation. These studies provided robust, contextually grounded evidence on digital literacy barriers experienced by older adults. The majority (n=13) of studies were appraised as moderate quality, most commonly due to limited reporting of researcher reflexivity, small or context-specific samples, and restricted transferability to more digitally excluded populations. Several review and cross-sectional studies relied on self-reported measures and cross-sectional designs, limiting causal interpretation of identified barriers. Studies rated as moderate-unclear quality (n=3) were retained in the review because these studies still contributed valuable descriptive and contextual insights relevant to understanding barriers among older adults.

### Primary Findings

Seven primary barriers of digital literacy among older adults were identified, including health barriers, support networks, convenience and ease of use, knowledge and information, perception barriers, resource barriers, and barriers for special populations. Within these 7 overarching themes, 18 associated categories were identified and provide a more detailed understanding of the specific challenges faced by older adults in adopting and using digital technologies. Consistent with scoping review methodology, the following sections thematically synthesize the 7 identified themes and categories, illustrating the diverse barriers to digital literacy among older adults.

### Health Barriers

Health-related barriers are a significant factor impeding older adults’ interaction with digital technologies. This theme encompasses 1 category that explores the physical and cognitive challenges faced by older adults when engaging with digital devices.

Evidence from the research shows older adults encounter significant health-related barriers that impede their interaction with digital technologies. Across studies, age-related physical, sensory, and cognitive changes (eg, vision impairment, reduced dexterity, memory challenges, fatigue, or chronic disease burden) limited older adults’ ability to learn, navigate, and sustain use of digital technologies [19-21,29,30,37,38]. In studies focused on digital health and self-management, chronic conditions compounded existing barriers by increasing cognitive load and task complexity, particularly where technologies required ongoing monitoring, interpretation of health information, and sustained engagement over time [24,35,40]. Cognitive impairments, such as memory loss and forgetfulness, were also frequently discussed barriers to digital literacy for older adults [34,35,38,39]. These barriers were frequently described as context-dependent, becoming more pronounced when digital tools were poorly aligned with aging-related needs or fluctuating health conditions, particularly in digital health and accessibility contexts [24,29,30,35,40].

### Support Networks

Support networks are essential in shaping older adults’ ability to engage with digital technologies. This theme explores 2 key categories: the impact of social support and social influence on digital literacy and technology adoption among older adults. While social support refers to the direct assistance and encouragement provided by family, friends, and community services, social influence encompasses the broader attitudes and behaviors within one’s social network that can shape technology use.

The studies reviewed highlight the crucial role that support networks play in helping older adults navigate the digital landscape. However, many older adults lack sufficient social support from family, friends, or community services, which exacerbates their digital literacy challenges [20,21,26,28,32-35,37,39]. Dependence on younger family members for learning new digital skills is not always reliable, as negative interactions, such as lack of patience, can reinforce feelings of inadequacy and discourage use [22,26,33,34,38]. The absence of family or social networks often results in older adults being less likely to adopt internet use [20,21,26,33,34].

Evidence from the reviewed literature shows that social influence is pivotal in the adoption of digital technologies among older adults. Positive or negative attitudes within social networks can impact older adults’ engagement with technology [19,24,29,34]. The encouragement or discouragement from family members and peers can either facilitate or hinder the adoption of digital tools [22,28,29,34,35,38].

### Convenience and Ease of Use

The convenience and ease of use of digital technologies are crucial factors that determine their adoption and sustained use among older adults. This theme is explored through two key categories: (1) the impact of application interface and design and (2) the challenges posed by continuous technological changes.

The studies reviewed underscore that the design and interface of digital applications are critical determinants of their usability for older adults. Complex interfaces, small font sizes, poor sound quality, and multiple authentication requirements pose significant challenges because they fail to account for the physical and cognitive changes associated with aging, such as declining vision, hearing loss, and reduced memory or cognitive processing speed [22,29,30,35,37,38,40]. Several studies noted that the cumulative burden of multistep processes (ie, repeated logins, verification requirements, and navigating across multiple platforms) further increased cognitive load and reduced sustained engagement among older adults [20,21,25,35,40].

Continuous updates and changes in digital devices and websites create additional hurdles for older adults, causing confusion and a loss of confidence in their ability to use these technologies [29,30,34,37,38]. The constant evolution of technology can be overwhelming and discouraging for older users, leading to reduced engagement.

### Knowledge and Information

The ability to effectively use digital technologies is heavily dependent on the knowledge and information available to older adults. This theme examines two key categories: (1) the impact of a lack of digital literacy and skills and (2) the consequences of limited awareness about digital technologies.

Research highlights that a pervasive lack of digital literacy among older adults is a significant barrier to the use of digital technology and services [19,20,22,25,26,33-40]. Many older adults did not grow up with advanced technology, making it challenging for them to acquire new digital skills [19,26,33,34,37,38]. This limited proficiency includes difficulty in navigating digital technologies, a lack of operational or technical knowledge, and minimal exposure to modern digital tools [20,22,27,33,34,37,38]. The general lack of computer proficiency hampers effective use of patient portals and other digital health platforms [25,28,31,35,40].

Many older adults are unaware of available digital technologies, services, and their benefits, which further limits their adoption of these technologies [19,20,27-29,33]. This lack of awareness hinders the effective use of digital health tools for managing health conditions. Beyond operational skills, several studies also reported difficulties appraising the credibility and relevance of online information, particularly in digital health contexts, which hindered confident use of digital services [20,21,25,31,35].

### Perception Barriers

#### Overview

*** ***Perceptions and attitudes toward digital technologies play a crucial role in shaping older adults’ willingness and ability to adopt and use these tools. This theme encompasses 4 key categories: concerns related to security, privacy, fear, and anxiety; challenges of self-efficacy and motivation; social and psychological barriers; and a preference for traditional methods over digital interactions.

#### Security, Privacy, Fear, and Anxiety

Concerns about online security and privacy are significant deterrents for older adults when considering the use of digital technology and services. Many older adults fear privacy breaches and distrust digital platforms, which prevent them from engaging with these technologies [19,21,27,29,32]. This fear is particularly pronounced among rural residents, who express significant worries about breaches of privacy and confidentiality, especially with technologies that monitor daily activities and collect personal data [35]. Beyond privacy concerns, the fear and anxiety older adults experience around technology use often stem from broader security issues. Reports of online scams have heightened these concerns, leading many older adults to avoid digital engagement and digital health services altogether [21,27].

In addition to privacy concerns, older adults often experience anxiety about constant monitoring and the implications of being watched or recorded without consent, further increasing their reluctance to engage with technology [29]. The mistrust of technology, coupled with the complexity of digital tools, exacerbates their anxiety, making digital engagement even more daunting [21,35]. Several studies also reported fear of making irreversible mistakes or damaging devices, which contributed to the avoidance of independent technology use [21,27,32,36,38,39].

#### Self-Efficacy and Motivation

Low self-efficacy and motivation significantly impact older adults’ engagement with digital technologies. Many older adults lack confidence in their ability to use telemedicine and other digital tools due to age-related issues, such as poor eyesight, memory loss, and unfamiliarity with technology [21,23,32,36]. Additionally, older adults’ beliefs that they are too old to learn new technologies or that digital tools are unnecessary for daily tasks further hinder their willingness to engage with these tools [23,35]. Internalized age-related stereotypes about technology use further undermined confidence, reinforcing beliefs that digital technologies were better suited to younger generations [19,23,26,34].

#### Social and Psychological Barriers

The adoption of digital technologies among older adults is often hindered by concerns related to social isolation, stigmatization, and threats to independence. Some older adults fear that reliance on smart technologies could lead to reduced human interaction and increased loneliness, with worries that increased digital interaction might replace physical activity and social engagement [27,29,36]. Additionally, the use of assistive technologies can sometimes lead to stigmatization, as older adults may fear being labeled as dependent or frail, which further discourages their use [29]. Concerns about personal autonomy are also prevalent, with older adults expressing hesitation toward technologies that might impose rigid structures or routines, thereby undermining their sense of independence [29].

#### Preference for Traditional Methods

Many older adults prefer traditional methods of communication, such as letters and in-person consultations, instead of digital interactions. They value the personal connection and the comprehensiveness of physical examinations, which they believe digital tools cannot replicate [19,29]. The study found a low demand for telemedicine services among rural residents, often due to a preference for face-to-face visits and discomfort with online consultations [29,35].

### Resource Barriers

*** ***Access to and the affordability of digital technologies are critical challenges that significantly hinder older adults’ ability to engage with digital tools. This theme explores 3 key categories: limited access to technology; financial constraints; and the lack of personalized training, all of which contribute to the digital divide among older adults.

Limited access to digital devices and the internet is a significant barrier for many older adults [27,37,39]. This includes issues such as unreliable internet access and lack of necessary equipment, including smartphones or computers [19,20,27,39,40]. Many rural residents particularly struggle with a lack of reliable internet access, which is critical for telemedicine adoption [39]. Not possessing smartphones or having limited access to reliable internet and other digital resources further exacerbates these challenges [20,27]. Residential facilities often lack the necessary infrastructure, hindering older adults’ ability to use digital health portals [27].

The affordability of digital devices and internet services is a substantial barrier. Many older adults have limited financial resources to purchase and maintain digital devices or pay for internet access [37,39,40]. The high cost of devices, maintenance, and indirect costs, such as electricity consumption, poses significant obstacles [39,40]. Lower income levels are associated with reduced internet use among older adults [20,21,24,26,40].

The absence of personalized training and ineffective teaching methods further hinder older adults’ ability to learn and use digital technologies by failing to accommodate their specific learning needs. Rigid and nontailored instructional approaches can lead to frustration, disengagement, and a lack of confidence, making the learning process overwhelming and exacerbating the digital divide [24,33,38,40].

### Barriers for Special Populations

#### Overview

In addition to the general barriers faced by older adults in adopting digital technologies, certain special populations encounter unique challenges that further complicate their ability to achieve digital literacy. Across studies, barriers affecting special populations were rarely experienced in isolation; instead, they reflected the interaction of health-related, perceptual, usability, and resource constraints, often compounding digital exclusion.

#### Visually Impaired Older Adults

Visually impaired older adults often lack awareness about the potential of technology to promote independence and reduce loneliness. This lack of awareness limits their engagement with digital tools that could otherwise enhance their quality of life. Additionally, municipalities frequently lack the expertise and capacity to provide adequate training, leaving visually impaired individuals without the necessary skills to use digital technologies effectively [30]. Another significant barrier for visually impaired older adults is the lack of long-term support after initial training. While some may receive training on how to use digital tools, the absence of continuous support, particularly when faced with technical problems or software updates, can result in the loss of newly acquired skills, thereby hindering sustained technology use [30]. Technological changes present a unique challenge for visually impaired older adults. Learning to use gesture-based interactions and screen readers requires extensive training. Frequent updates and the presence of inaccessible content further complicate the use of digital devices, making it difficult for visually impaired individuals to keep up with technological advancements [30]. These challenges were frequently attributed to inconsistent accessibility standards and design practices, rather than individual limitations, and often resulted in frustration and the abandonment of digital technologies.

#### Older Adults With Chronic Conditions

Older adults with chronic conditions experienced increased digital literacy barriers due to the combined demands of health self-management and technology use. Studies reported that symptom burden, fatigue, and fluctuating health status increased cognitive load, making it difficult to learn, interpret, and sustain engagement with digital health tools. Technologies requiring frequent monitoring, data interpretation, or consistent interaction were particularly challenging, often leading to reduced confidence and disengagement [24,35,40].

#### Older Adults With Cancer and Their Caregivers

Both older adults with cancer and their caregivers often lack confidence in their ability to find relevant health information online. This low self-efficacy acts as a barrier to the effective use of digital health tools [34]. Limited familiarity with digital tools and apps is common among older adults with cancer and their caregivers. Additionally, lower levels of education are associated with lower eHealth literacy, making it more challenging for these individuals to engage with digital health resources effectively [31]. The rapid evolution of digital tools creates a barrier for older adults with cancer and their caregivers, who may struggle to keep up with technological changes. This unfamiliarity with new tools can prevent them from fully utilizing digital health resources [31]. Finally, limited access to necessary resources, such as broadband, computers, and other digital devices, particularly in rural areas, further exacerbates the digital divide for older adults with cancer and their caregivers. This geographic disparity highlights the need for targeted interventions to improve digital access and literacy in underserved areas [31].

#### Culturally and Linguistically Diverse Older Adults

Culturally and linguistically diverse older adults experience distinct digital literacy barriers related to language proficiency, cultural relevance, and reliance on informal support networks. Limited English proficiency and unfamiliarity with technical and health-related terminology restrict the understanding of digital platforms, online instructions, and digital health information, even when access to technology is available [26,28]. These barriers are often compounded by limited access to culturally appropriate training and support, resulting in greater dependence on family members or community intermediaries for navigation and interpretation of digital tools [28]. In some cases, this reliance reduced autonomy and confidence, further discouraging independent technology use. Cultural perceptions of technology, trust, and privacy also shaped engagement, highlighting the need for culturally responsive approaches to digital literacy support [26].

### Defining Digital Literacy

In addition to the 7 themes identified in this scoping review, the included studies demonstrated heterogeneous definitions of digital literacy, underscoring the absence of a unified conceptualization in the field. Given this variability, we did not impose a single operational definition for the purposes of synthesis. Instead, we documented and compared the range of conceptualizations described in the included studies to accurately reflect the existing heterogeneity in the literature. Aslan et al [19] define digital literacy as the ability to find, evaluate, and communicate information using information and communication technologies, emphasizing the core skills necessary for effective information management. Kebede et al [20] offer a comprehensive framework, outlining digital literacy as comprising 5 key indicators: information and data literacy, communication and collaboration, digital content creation, safety, and problem-solving. This structured approach reflected the multidimensional nature of digital literacy. Chee [27] focuses on the practical application, defining digital literacy as the ability to use devices and online platforms, which highlights the functional aspect of digital literacy. Finally, Baek et al [22] define digital literacy as the ability to use and understand information obtained from various digital devices, stressing the significance of comprehension within the digital literacy framework.

## Discussion

### Principal Findings

This scoping review identified 7 interrelated domains of barriers to digital literacy among older adults: health barriers, support networks, convenience and ease of use, knowledge and information, perception barriers, resource barriers, and barriers for special populations. Together, these themes capture a broad range of challenges, such as physical and cognitive impairments, lack of social support, complex app interfaces, limited digital literacy, security concerns, financial constraints, and a preference for traditional methods of communication. These findings highlight the intricate and multifaceted nature of the digital divide among older adults.

While digital literacy and technology adoption barriers have often been reported separately, recognizing their complex interplay is crucial. Prior research has often examined digital literacy barriers in isolation, focusing separately on technology adoption, access, or specific older adult populations [7-12]. This synthesis suggests that older adults frequently encounter multiple barriers simultaneously, aligning with ecological and life-course perspectives on technology use in later life. This pattern is consistent with ecological and life-course models of aging and technology use, which emphasize how individual, social, and environmental factors interact over time to shape engagement with digital technologies in later life [42-44]. Many of these barriers do not exist in isolation; rather, they often work together to create compounded difficulties for older adults. For instance, limited access to technology can exacerbate feelings of anxiety and insecurity, while financial constraints may prevent access to personalized training that could alleviate these concerns [20,21]. Similarly, social isolation may intensify the impact of low self-efficacy, leading to a reluctance to engage with digital tools [24,30,38]. An example of this interplay can be seen in how physical barriers, such as poor motor skills or visual impairments, can exacerbate challenges related to convenience and ease of use [30,31,42,43]. Complex interfaces or small font sizes can make digital devices particularly difficult for those with physical limitations, further discouraging use [23,30,31,43,45]. Therefore, it is important to view digital literacy barriers holistically, understanding that older adults often experience multiple, interconnected challenges that influence their ability to use and benefit from digital technologies. Addressing these barriers effectively requires an integrated approach that considers the full spectrum of factors influencing digital literacy.

These findings extend the literature on digital literacy among older adults by synthesizing a wide range of reported barriers and demonstrating how they cluster across multiple domains, including resource constraints, perceptions of technology, and social and psychological factors. Rather than occurring in isolation, barriers were frequently described as interconnected, with multiple challenges shaping older adults’ experiences of digital engagement. This synthesis supports a more nuanced understanding of digital literacy that moves beyond single-factor explanations and highlights the cumulative and interacting nature of barriers encountered in later life.

In relation to eHealth technologies, the findings highlight how barriers commonly associated with eHealth literacy, such as design complexity, cost, and limited awareness, are frequently described alongside broader influences, including social support and perceptions of technology [5,19-21,39]. This pattern suggests that challenges related to eHealth engagement are embedded within wider digital literacy contexts rather than being solely attributable to individual skill deficits. Consistent with broader eHealth literacy literature [ie, 45,46], these findings suggest that engagement with digital health technologies is shaped by intersecting usability, access, and psychosocial factors rather than individual competence alone. Taken together, these observations point to the importance of considering multiple, interacting factors when examining eHealth literacy among older adults.

### Defining Digital Literacy

Despite the increasing recognition of digital literacy as a critical competency for older adults, this review identified substantial variability in how digital literacy is defined and operationalized across studies. Definitions ranged from basic functional skills to more comprehensive frameworks encompassing information evaluation, communication, safety, and problem-solving. This heterogeneity complicates cross-study comparison and limits the development of targeted interventions.

Consistent with prior work, including the review by Oh et al [47], many commonly used measurement tools emphasize narrow aspects of digital literacy while overlooking broader competencies such as digital content creation and online safety. Although frameworks, such as the digital competence model, propose multidimensional conceptualizations, few studies apply these comprehensively in research with older adults. The lack of comprehensive assessment tools limits the ability to accurately evaluate digital literacy and develop targeted interventions.

The absence of a universally accepted definition of digital literacy complicates research efforts and impacts how digital literacy is assessed across studies. Varying definitions contribute to fragmented approaches, with some emphasizing technical proficiency, while others incorporate cognitive and social dimensions. This inconsistency leads to measurement gaps, as most widely used tools fail to capture the full scope of digital skills needed for meaningful engagement with technology. For instance, while some assessments, such as the Mobile Device Proficiency Questionnaire, evaluate all 5 digital competence competencies, many omit crucial aspects, such as digital content creation and online safety [47]. Without a standardized framework, it is difficult to compare findings across studies or develop interventions that effectively address older adults’ digital literacy needs [47].

### Future Directions

To address conceptual variability in the literature, future research would benefit from explicitly defining how digital literacy is conceptualized within the context of older adulthood or, at minimum, clearly acknowledging heterogeneity in existing definitions when designing studies. Greater conceptual clarity, supported by the development and adoption of standardized and validated measurement frameworks, would enhance the comparability and coherence of findings across studies. Establishing a shared foundation for defining and measuring digital literacy would also strengthen policy and practice efforts by ensuring that digital inclusion initiatives align with the real-world needs of older adults [47].

Future research should prioritize the development and evaluation of tailored interventions that address the specific barriers identified in this review [25,37]. Longitudinal studies are needed to assess the sustained impact of digital literacy programs on older adults’ quality of life and health outcomes. Interdisciplinary research that integrates gerontology, technology design, and education can offer a more comprehensive approach to enhancing digital literacy among older adults. Collaboration with community organizations, policymakers, and technology developers will be essential in designing and implementing effective, scalable interventions.

Additionally, researchers should examine the barriers faced by specialized populations, such as older adults with visual impairments, chronic conditions, and those from rural or low-income backgrounds. These groups encounter unique challenges that often amplify difficulties in engaging with digital technologies. For instance, older adults with visual impairments struggle with the lack of accessible content and frequent technological changes, which can render screen readers and gesture-based interactions ineffective without extensive training [31]. Addressing these specialized needs through inclusive technology design and tailored interventions is crucial to ensure that all older adults can achieve digital literacy and benefit from technological advancements.

This review highlights the need for multifaceted interventions that simultaneously address the interconnected barriers older adults face [20,21,25,30]. Future studies should explore approaches that integrate digital literacy training with accessible and affordable technology solutions. Additionally, research should evaluate the effectiveness of personalized and community-based approaches to digital literacy, particularly those that involve collaboration with educators, social workers, and family members. Investigating these strategies will provide evidence-based recommendations for scalable interventions that not only improve digital literacy among older adults but also enhance their overall quality of life.

### Practical Implications

The findings of the scoping review have several practical implications for policymakers, practitioners, and technology developers. By highlighting the interconnected nature of digital literacy barriers, this review can support the development and funding of comprehensive interventions that address multiple challenges simultaneously, rather than focusing on isolated barriers. Policymakers should consider creating and funding programs that increase the accessibility and affordability of digital devices and internet services for older adults. Consistent with the findings of this review, affordability remains a significant barrier, with many older adults unable to purchase or maintain digital devices or pay for reliable internet access [20,21,25,27,45]. Addressing cost-related barriers is a necessary foundation for improving digital participation among older adults, particularly those living on fixed or limited incomes.

Practitioners can leverage insights from this review to design and implement community-based digital literacy programs that address the diverse and intersecting needs of older adults. The results highlight the importance of personalized, ongoing, and confidence-building approaches to training, particularly for individuals with limited prior exposure to digital technologies or reduced self-efficacy [25,37]. 

Technology developers play a critical role in reducing digital exclusion by prioritizing user-centered designs that accommodate age-related sensory, cognitive, and motor changes. The findings emphasize the need for intuitive interfaces, simplified navigation, and accessible design features, such as readable text, clear audio, and reduced cognitive load [23,30,31]. In addition, providing continuous education, guidance, and support related to technological updates can help older adults maintain confidence and sustained engagement, particularly as frequent changes were shown to undermine usability and self-efficacy [21,30,31,38]. By implementing these coordinated strategies, policymakers, practitioners, and technology developers can work together to address the multifaceted barriers identified in this review and support more equitable digital participation among older adults.

### Limitations

Several limitations of the scoping review should be acknowledged. First, the exclusion of non-English studies and older publications may have limited the scope of insights into global digital literacy barriers among older adults. However, this decision was made to ensure consistency in data interpretation and to focus on more recent studies that reflect the rapidly evolving digital landscape. Additionally, this review focused solely on the experiences of older adults, excluding perspectives from individuals who play a key role in digital literacy education, such as tutors, librarians, and other educators. This approach was chosen to center firsthand accounts of the barriers faced by older adults. While the perspectives of educators and other stakeholders are undoubtedly valuable, the scope of this review was intentionally narrowed to prioritize firsthand accounts from the target population. As such, future research should consider incorporating educators’ insights to provide a more comprehensive understanding of the barriers to digital literacy among older adults, ultimately informing more effective, tailored interventions.

### Conclusion

The scoping review provides a comprehensive synthesis of the key barriers to digital literacy among older adults, highlighting the intricate and multifaceted nature of these challenges. The review identified 7 primary themes (ie, health barriers, support networks, convenience and ease of use, knowledge and information, perception barriers, resource barriers, and barriers for special populations) that collectively shape the digital experiences of older adults. These barriers often interact, potentially increasing the challenges older adults face in engaging with digital technologies. These findings suggest that engagement with digital technologies is shaped by a complex interplay of individual, social, and contextual factors. Future work could explore strategies that address these barriers while considering diverse abilities, levels of support, and contextual constraints. Additionally, research would benefit from greater consistency in defining digital literacy and examining how multifaceted approaches may support digital participation among subpopulations facing heightened barriers.

## Supplementary material

10.2196/80647 Multimedia Appendix 1 Search strategy.

10.2196/80647 Multimedia Appendix 2 Included study characteristics [19-40].

10.2196/80647 Checklist 1 PRISMA-ScR checklist.

## Abbreviations

ICT: information and communication technologies
JBI: Joanna Briggs Institute
MeSH: Medical Subject Headings
PRISMA: Preferred Reporting Items for Systematic Reviews and Meta-Analyses
PRISMA-ScR: Preferred Reporting Items for Systematic Reviews and Meta-Analyses extension for Scoping Reviews
