# Supplementary material for: Key Challenges and Barriers to Digital Literacy for Older Adults: Scoping Review
Source: JMIR Aging. 2026 Mar 16;9:e80647. doi: 10.2196/80647 (PMC12991319; doi:10.2196/80647)
Supplement: Multimedia Appendix 1 [file aging-v9-e80647-s001.docx]

**Search Strategies**

**Database:** PubMed

**Platform:** National Library of Medicine

**Date searched:** June 18, 2025 (initial) and December 15, 2025 (updated search)

**Time span:** January 2014 to December 2025

**Language** **limits**: English

**Document** **types**: Articles and reviews

**Records Retrieved:** n = 388 (initial search), n = 123 (updated search), total n = 511

**Search: (**digital literacy OR digital divide OR digital inclusion OR computer literacy) AND (barriers OR challenges) AND (older adults OR elderly OR seniors)

**Database:** MEDLINE(R)

**Platform:** Ovid

**Date searched:** June 18, 2025 (initial search) and December 15, 2025 (updated search)

**Time span:** January 2014 to December 2025

**Language** **limits**: English

**Document** **types**: Articles and reviews

**Records Retrieved:** n = 186 (initial search), n = 15 (updated search), total n = 201

**Search:** (older adults OR aged OR aged, 80 and over, OR seniors) AND (digital literacy OR computer literacy OR digital divide OR digital inclusion) AND (barriers OR challenges)

**Database:** Web of Science Core Collection

**Platform:** Clarivate Analytics

**Date searched:** December 15, 2025

**Time span:** January 2014 to December 2025

**Language** **limits**: English

**Document** **types**: Articles and reviews

**Records Retrieved:** n = 786

**Search:** (ALL=(digital literacy OR digital divide OR digital inclusion OR computer literacy OR internet literacy) AND (barriers OR challenges) AND (older adults OR seniors OR elderly)

**Database:** EBSCOhost (All Databases)
**Platform:** EBSCOhost
**Date searched:** December 15, 2025
**Time span:** January 2014 to December 2025
**Language limits:** English
**Document types:** Articles and reviews
**Records retrieved:** n = 250

**Search:** (Digital literacy OR digital divide OR computer literacy OR technological literacy OR internet literacy OR digital inclusion) AND (challenges OR barriers) AND (older adults OR elderly OR seniors)
