# Supplementary material for: Key Challenges and Barriers to Digital Literacy for Older Adults: Scoping Review
Source: JMIR Aging. 2026 Mar 16;9:e80647. doi: 10.2196/80647 (PMC12991319; doi:10.2196/80647)
Supplement: Multimedia Appendix 2 [file aging-v9-e80647-s002.docx]

**Included Study Characteristics**

| **Study** | **Methods** | **Population** | **Country** | **Key findings related to barriers to digital literacy** |
| --- | --- | --- | --- | --- |
| Aslan et al. [19] | Meta-ethnography of qualitative studies | Older Adults Aged 60 or 65+ years old (as defined in included studies) | Multiple Regions (studies from Europe, North America, Australia) | Specific to digital literacy, may older adults state their digital literacy levels were low, and were influenced by various barriers. This included preference for using the telephone or arranging face-to face appointments, not growing up with advanced technology, belief they were “too old” to learn digital skills, or belief that it is too difficult to understand how to use digital services. Barriers related to older adults’ capacity to understand digital technologies included individual frustration, lack of user manuals with new technologies, fear of making mistakes, or physical impairment impacting the physical use of devices. |
| Kebede et al. [20] | Scoping review | Studies with participants being older adults with mean age 65+ years old | International | Barriers to older adults’ **ability to find, understand, evaluate, and use digital technologies.** Individual barriers include limited digital skills and knowledge, low confidence, cognitive or physical limitations, and fear or frustration with technology. Technological barriers relate to complex, poorly designed, or rapidly changing devices and interfaces, as well as accessibility and usability issues. Social and cultural factors include lack of support, social isolation, stigma, and cultural expectations, while structural and environmental barriers include limited access to devices or internet, financial constraints, and inadequate training or guidance. Together, these factors reduce motivation, perceived relevance, and ability to engage effectively with digital technologies. |
| Money et al. [21] | Qualitative interviews | Older adults aged 75+ years old | United Kingdom | The barriers to digital technology use among older adults included fear of scams and misuse of personal information, lack of ongoing support for learning or maintaining skills, and a preference for face-to-face interactions. Many also perceived difficulties with technology as typical for their generation. Additionally, some saw the convenience and easy access provided by digital technologies as a barrier, as it could reduce cognitive engagement, physical activity, and social interaction. |
| Baek et al. [22] | Qualitative interviews | Older adults aged 65+ years old | South Korea | Participants identified several contextual barriers to digital literacy, including age-related physical and cognitive changes (such as vision problems, physical discomfort, forgetfulness, and slower learning), limited opportunities to learn digital skills, and reduced social contact during the COVID-19 pandemic. Family and peer support played a mixed role: while the absence of close or supportive family members was often a barrier, some participants also experienced discouragement or over-assistance from relatives, which limited their opportunities to learn independently. |
| Köttl et al. [23] | Qualitative interviews | Participants aged between 69 and 88 years old | Multiple Regions (Austria and Israel) | Engagement with everyday information and communication technology was limited by internalized age stereotypes, low perceived relevance of digital technologies in later life, and beliefs that declining competence and learning ability are a normal part of aging. Barriers were further reinforced by usability and design challenges, fear of making mistakes, and limited or discouraging intergenerational support. Structural factors such as rapid technological change, inaccessible training opportunities, and increasing reliance on online services also contributed to disengagement and non-use. |
| Chang et al. [24] | Quantitative cross-sectional survey | Older adults 60+ years old | United States | Barriers to digital literacy among non-Internet users were most commonly related to lack of knowledge on how to use the Internet, with approximately half indicating a need for training or support. Mistrust of the Internet, particularly concerns about privacy and security, was the next most frequently cited barrier, followed by limited access, cost, fear of use, physical limitations, time demands, and language challenges. Despite these barriers, most non-users did not perceive their non-use as problematic, reporting that they did not feel they missed important information or were pressured to go online. |
| Low et al. [25] | Qualitative interviews | Older adults 50+ years old | Singapore | Barriers among older adults included lack of access to smartphones, limited affordability of devices, applications, and third-party services, and usability challenges such as small text, complex interfaces, and difficulty typing. Privacy and data protection concerns, particularly fear of information misuse, further hindered engagement. Physical limitations (e.g., vision impairments) and low perceived need or motivation also contributed to non-adoption, despite general openness to using simple, useful technologies for health management when barriers were minimized. |
| Tan & Chan [26] | Qualitative interviews and focus groups | older adults aged 55 to 75 years old | Singapore | Use of ICT among participants was shaped by fear and anxiety related to limited English proficiency, low education, and financial concerns. Past negative experiences with technology contributed to feelings of inadequacy and emotional stress. Family influence played a key role, with encouragement facilitating ICT use and discouragement limiting access and confidence. Social reinforcement through peers and networks further shaped attitudes toward ICT, particularly among those with higher socioeconomic status. |
| Chee [27] | Qualitative interviews and phenomenology approach | Older adults 60+ years old | Malaysia | Use of digital technologies was constrained by limited access to devices and reliable internet, low digital skills due to minimal prior exposure, and language barriers that hindered understanding of online content. Participants also reported fear of online scams, privacy concerns, and low trust in digital platforms, alongside usability challenges such as small text, complex interfaces, and unfamiliar terminology. Age-related functional limitations (e.g., vision impairment, arthritis) and internalized beliefs that technology is too complex or irrelevant in older age further contributed to disengagement. |
| Hyman et al. [28] | Qualitative participatory community participatory action methodology with focus groups and photovoice methods | Individuals over the age of 18 years old, with a subset population of older adults 50 years and older | Canada | In this study, adults aged 50 and older identified several barriers to using digital health tools, including limited digital literacy, older age–related difficulties learning new technologies, and lower levels of formal education. Language barriers further hindered access to and understanding of digital content, while concerns about trust, accuracy, and privacy led many to prefer information from healthcare professionals rather than online sources. Lack of training, ongoing support, and time to learn also reduced confidence and engagement with digital technologies. |
| Street et al. [29] | Qualitative World Café forums | Older adults aged 55+ years old | Australia | The study identified several concerns related to digital literacy among older adults, including difficulty understanding complex technologies, feeling overwhelmed by rapid technological change, and low confidence in their ability to learn and use new digital tools. Participants also described a gap between having access to technology and being able to use it meaningfully, as well as a mismatch between technology design and their abilities or needs. These factors contributed to frustration, disengagement, and limited adoption of digital and smart technologies. |
| Fuglerudet al. [30] | Qualitative interviews and stakeholder meetings. | Visually impaired older adults 60+ years old | Norway | Older adults with visual impairment faced barriers to digital literacy primarily related to limited access to specialized, individualized training and insufficient capacity and expertise within municipal services. Long waiting times, lack of ongoing support and follow up, low awareness of training entitlements, and reliance on sighted relatives for digital tasks hindered the development and maintenance of digital skills. |
| Verma et al. [31] | Scoping review | Older adults 65+ years old living with cancer or cancer survivors and their caregivers | International | eHealth literacy among older adults was limited by both intrinsic and extrinsic barriers. Intrinsic barriers included low confidence, difficulty evaluating information quality, limited familiarity with digital tools, and challenges applying online information to personal health needs. Extrinsic barriers included rapid technological change, limited access to devices and broadband, and lower educational attainment. Geographic remoteness and infrequent use of digital health resources further reduced effective engagement with eHealth tools. |
| Balci & Aslan [32] | Qualitative interviews | Older adults 65+ years old | Turkey | Three themes emerged from the analysis: purposes of using digital health technologies, barriers to adoption, and perceived benefits. Participants reported several barriers, including lack of digital literacy, perceptions of being too old to learn, age-related changes, economic constraints, dependence on family support, and concerns related to digital technologies. |
| Rojas [33] | Qualitative interviews | Older adults 60+ years old | Costa Rica | The main observed barriers for learning about and using Information and Communication Technologies (ICT) were lack of experience with ICT, negative emotions such as fear, and shame related to the learning process and restrictions for individual support. On the other hand, the main supportive factors for ICT learning were a collaborative learning environment, the motivation for becoming efficient and independent ICT users, and using learning strategies focused on supporting cognitive abilities and overcoming lack of experience with ICT. The most observed factors for overcoming the identified barriers included peer support, the motivation for using ICT independently, and the opportunities for practicing the learned skills. |
| Schreurs et al. [34] | Qualitative discourse analysis | Older adults | Canada | • “Digital literacy paradox” (access ≠ empowerment)  • Tension between independence and coercive digitalization  • Media narratives shaping self-perception |
| Shao et al. [35] | Descriptive qualitative approach with semi-structured interviews | Older adults 60 years and older | China | Constraints to digital health literacy included cognitive blind spots, misunderstanding biases, poor basic skills and challenges in digital adaptation,  psychosocial limitations such as multiple role conflicts, social image concerns, and health information avoidance, issues with health information quality, and concerns about digital security risks. |
| Fernandez-Piqueras et al. (36) | Quantitative non-experimental cross-sectional design with surveys | Older adults 65+ years old | Spain | The study identified key barriers to digital literacy among older adults, including low competence in online collaboration, basic problem solving, and networking. Older participants and those with lower professional qualifications were more likely to demonstrate consistently low digital skills across all domains, indicating structural and skills-based barriers to digital literacy. |
| Gitlow (37) | Quantitative nonexperimental survey | Older adults 65+ years old | United States | The primary barriers to digital literacy among older adults were a combination of skill- and ability-related factors, including lack of knowledge or technical skills, difficulty navigating programs, memory or cognitive challenges, vision impairments, and fine motor difficulties. In addition, many older adults reported a perceived lack of need or interest in using technology, which limited their engagement with devices such as cell phones, computers, tablets, and e-readers. |
| Vaportzis et al. (38)_ | Qualitative focus groups | Older adults 65 to 76 years old | United Kingdom | The focus groups identified several barriers among older adults, including lack of instructions or guidance, low knowledge and confidence, health-related challenges (e.g., vision, arthritis, dexterity), and cost of devices. Additional concerns included technology being too complex or overwhelming, feelings of inadequacy compared with younger generations, and difficulty with tablet features such as small buttons or device weight. |
| Baker et al. (39) | Qualitative Action Research with interviews, reflective journals and digital artefacts | Older adults between 58 to 81 years old | Australia | The barriers identified include **technological difficulties**, such as confusing user interfaces, unintuitive applications, accidental disabling of devices, and challenges navigating essential online services; **geographic and economic barriers**, including poor internet connectivity in rural areas and costly data plans; and **social disadvantage**, where participants’ social isolation and limited real-life networks made it difficult to build online connections and practice ICT skills. Additional factors such as low literacy, health issues, and lack of prior experience further compounded these challenges. |
| Cajita et al. (40) | Qualitative interviews | Older adults 65+ years old | United States | Across these findings, barriers to digital literacy among older adults included lack of knowledge and technical skills, which often led to fear of making mistakes and reluctance to engage with digital tools, as well as difficulty keeping pace with rapidly changing technologies. Sensory limitations such as reduced vision or hearing, poorly designed interfaces with small icons or low contrast, and perceived high costs, particularly for those on fixed incomes, further constrained use. Some participants also reported a low perceived need for technology, reducing motivation to develop digital skills in the absence of clear benefits or adequate training. |
